# Substance use among helping professionals: rationalization and negotiation of professional identity

**DOI:** 10.3389/fpsyt.2026.1953018

**Published:** 2026-10-26

**Authors:** Gabriel Kwun Wa Lee, Gloria Hongyee Chan, Yiu Tsang Andrew Low, T. Wing Lo

**Affiliations:** 1 City University of Hong Kong, Hong Kong, Hong Kong SAR, China; 2 Saint Francis University, Hong Kong, Hong Kong SAR, China

**Keywords:** cognitive dissonance, helping professionals, identity struggles, rationalization, rehabilitation, substance use

## Abstract

**Introduction:**

Given the scarce research on the drug use situation among helping professionals and the existing organizational structures that may affect them, this study explores how helping professionals in Hong Kong make sense of personal drug use and negotiate professional identity.

**Methods:**

Narrative, semi-structured interviews were conducted with 15 helping professionals. Interviews were conducted in Cantonese, transcribed verbatim, and translated into English, prioritizing semantic equivalence for colloquialisms. Culturally specific terms were retained in parentheses with brief explanatory notes where needed. An interpretivist, reflexive thematic analysis was undertaken using NVivo.

**Results:**

Drug use served coping, social and/or sexual functions. Participants managed cognitive dissonance through compartmentalization, normalization and critiques of illegality, and by reframing lived experience as strengthening rapport and credibility with clients.

**Conclusion:**

While their drug use may not have directly caused impairment, their rationalization of behavior instead of disclosing it and seeking professional support suggests underlying organizational and institutional issues. Anticipated workplace sanctions and professional impairment discourse fostered concealment and delayed help-seeking. Therefore, rather than adopting a zero-tolerance governance approach, establishing clear practice-related thresholds and providing confidential, harm reduction-informed workforce supports may better protect these professionals’ wellbeing and ensure service quality.

## 1 Introduction

The number of reported substance users in Hong Kong dropped from 8,777 in 2015 to 5,068 in 2024 (1). However, some specific substance-using groups may not have been reported, such as helping professionals who use Substances. While no formal research has been conducted in Hong Kong to investigate this phenomenon, it has been stated in news reports and official documents that doctors took cannabis (2), whilst some social workers took sedatives to alleviate their psychological distress (3). Existing literature from other countries indicates that helping professionals experience fatigue due to erratic working hours and work stress, leading them to use Substances for relief and wakefulness (e.g., 4, 5). They may also experience identity struggles as both practitioners and substance users, and unsuccessful resolution of these struggles may cause further distress, hampering their professional practice (e.g., 6–8). This suggests that helping professionals may contend with mental health and behavioral issues, highlighting the need for specific support.

In highly regulated helping professions, substance use is frequently framed through professional impairment discourse and organizational risk management, which can intensify stigma and encourage concealment rather than early help-seeking (e.g., 6, 9, 10). When practitioners anticipate disciplinary action, mandatory reporting, or other workplace sanctions, support needs may remain hidden until risks escalate (11–13). Given this context, examining how helping professionals who engage in substance use make sense of their behavior and negotiate professional identity is directly relevant to drug policy, workforce governance, and service quality implications.

Helping professionals are not immune to substance use, and emerging evidence highlights both its prevalence and potential contributing factors. While the number of helping professionals who engage in substance use is unknown in Hong Kong due to insufficient research, relevant studies from other countries shed some light on the prevalence of this issue. It has been noted that around 8%–15% of doctors, nurses and pharmacists have engaged in substance use (e.g., substances, alcohol) at work (9, 14, 15). A survey of 5,597 social workers conducted by Straussner and colleagues in 2015 found that nearly 9.5% and 5.7% had problems with alcohol and substances, respectively, and that 9.7% had alcohol and substance problems before becoming social workers and 7.7% of them had such problems throughout their social work career (16). This shows that despite their professional image and qualifications, they are still susceptible to substance use, similar to the general public (10, 17), and this warrants attention.

Burnout is commonly conceptualized as emotional exhaustion, depersonalization, and reduced personal accomplishment (18). In high-demand helping roles, burnout and distress may increase the likelihood of substance use as short-term coping (e.g., 5, 19).

The impact of substance use among helping professionals extends beyond their personal well-being to their capacity to practice safely and ethically. Substance use among professionals can lead to impairment, meaning an inability to provide care and up-to-standard practice (20). For instance, nurses who take substances experience “cognitive failure” at work, characterized by “errors in perception, memory, or action” (21, pp. 2, 8). There are social workers who use substances reporting that such behavior influences their decision-making and their engagement with clients (22). It may pose risks or harm to service users, violate the code of conduct, and even jeopardize the reputation of the helping profession (e.g., 6, 9, 23).

Since the identity of being a substance user conflicts with the identity of being a helping professional (16, 24), they may experience internal struggles or conflicts. These identity struggles can be illustrated in terms of cognitive dissonance. Cognitive dissonance refers to the mental discomfort an individual experiences when holding contradictory beliefs or values (25). For helping professionals who are also substance users, this dissonance arises from engaging in behaviors they are trained to prevent (26). Aware of the risks associated with substance use, these professionals face a conflict between their professional beliefs and personal behaviors, leading to significant dissonance.

There are various strategies for reducing dissonance, including attitude change, distractions and forgetting, trivialization and self-affirmation, denial of responsibility, development of consonant cognitions, behavior change, and rationalization (27). McGrath (27) reviewed various modes of dissonance reduction and concluded that the development of consonant cognitions and rationalization is a typical response to dissonance when the beliefs and values are firmly advocated. With their professional identity and knowledge, helping professionals who use substances are confident in their capability to use substances in a restrained manner, manage the effects of substances, and maintain their daily lives (13, 28). Therefore, they likely adopt consonant cognitions and rationalize their substance use.

Despite the existence of rehabilitation program for these professionals (e.g., Greenhouse Recovery Center) (29), they may not seek help due to the following factors:

Regarding internal factors, helping professionals often face fear, embarrassment, and psychological difficulties when seeking help for their substance use issues, and they tend to minimize or deny the severity of their issues (5, 12, 13). Given the positive public image and even idealization of these helping professionals, a greater degree of stigma, shame, and fear was identified when their substance use was noted (8, 12, 30). Zerubavel and Wright (31) noted that therapists who wished to openly share their psychological pain or wounds were stigmatized and excluded from getting support. Due to the punitive consequences, such as licence suspension or revocation, job loss, legal consequences and a lack of support, helping professionals who use substances tend to keep their substance problems secret, not revealing it to their employers/regulators (5, 9–12, 32). Furthermore, helping professionals who engage in substance use often exhibit low motivation to seek help. Warren et al. (13) outlined that some helping professionals who engage in substance use trivialize their problematic behavior as self-manageable or view their ordinary colleagues as inept in facilitating improvement. In particular, doctors tend to have high self-esteem rooted in their professional identity and knowledge, which may lead them to believe that they can manipulate the effects of substances and be immune to the harms associated with substance use (28).

External factors may play a larger role in affecting the motivation to receive treatment. Studies have uncovered that ordinary helping professionals likely hold negative attitudes towards substance users (e.g., disrespect, resentment, and even discrimination), leading to treatment failures and a feeling of disempowerment (33–36). This atmosphere further prohibits the intention of substance-using helping professionals to confess their problematic behaviors. Even if they seek treatment, they prefer individual or confidential treatment and reject group-based intervention (28).

Hong Kong adopts a multimodality approach to treatment and rehabilitation, which requires collaboration among various helping professionals (e.g., counselors and medical professionals) to achieve abstinence (37). This shows that helping professionals are essential in the rehabilitation of substance users. However, knowledge about helping professionals who are substance-using is largely in the shadows, particularly in the Hong Kong context. Such a phenomenon constitutes a complex and challenging issue for the field of rehabilitation, as these professionals are expected to serve as role models and provide support for their clients (e.g., 13, 16). Against this backdrop, this study investigates the phenomenon of substance use among rehabilitation professionals, including the type of substances they use and the reasons behind their usage, as well as their in-depth experiences, inner sense, and perspectives regarding their identity and how they navigate identity struggles. It is expected that this study can enhance the understanding of substance use in the helping professions and provide valuable insights for policy and practice.

## 2 Materials and methods

Given the limited existing research on substance use among helping professionals in Hong Kong, this exploratory study does not seek to establish the prevalence, clinical severity, or objective consequences of such substance use, but rather to examine how a largely under-researched group understands, interprets, and makes sense of its experiences in relation to professional identity and practice.

In this study, helping professionals refer to practitioners whose roles involve providing care, support, treatment, rehabilitation, education, or counseling to people at risk of, or affected by, substance use. We use the term “helping professionals who use substances/engage in substance use” to mirror terminology used in parts of the international literature on professional health programs. However, this study focuses on participants’ self-reported substance use and related experiences and should not be interpreted as a clinical assessment of impairment or fitness to practice.

### 2.1 Sampling

Purposive sampling was used to select participants who could provide rich, detailed, and relevant data to meet the study’s objectives. Eligible participants were helping professionals involved in substance-use prevention, rehabilitation, education, healthcare, or related services who also self-reported personal substance use (38). Participants might not take the core role in substance use prevention and rehabilitation, but were at least responsible for the education, rehabilitation, and health care for substance users. Recruitment was conducted through the Internet (i.e., Instagram pages, Facebook, and Telegram groups), personal networks, and assistance from welfare organizations.

Despite the difficulty of recruiting helping professionals willing to disclose their substance-use issues while still working in the field, the present study managed to recruit 15 participants aged between 21 and 37. All participants were Hong Kong citizens. Participant characteristics are presented in aggregate in **Table 1** to describe the sample while reducing the risk of deductive identification.

**Table 1:** Sociodemographic characteristics of participants.

Characteristic		n	%
Sex	Male	9	60
	Female	6	40
Age range	21–30 years old	11	73.3
	31–40 years old	4	26.7
Sexual orientation	Heterosexual	12	80
	Homosexual	3	20
Professional background	Medical/health professional	6	40
	Social work	4	26.7
	Education	4	26.7
	Law enforcement	1	6.7
Age at first substance use	Range	18–32	/
	Median	21	/
Type of substance use *	Cannabis	14	93.3
	Ecstasy/MDMA	5	33.3
	Crystal methamphetamine (Ice)	4	26.7
	LSD	4	26.7
	Ketamine	2	13.3
	Alkyl nitrites (Poppers)	2	13.3
	GHB	1	6.7
	Cocaine	1	6.7
	Nimetazepam (Erimin 5)	1	6.7

“Type of substance use” refers to lifetime self-reported substance use.

### 2.2 Data collection

To achieve the research objectives, the interview format was semi-structured narrative interviews, while the analysis was thematic. All interviews were conducted in Cantonese. Interviews lasted approximately 1.5 hours and focused on participants’ substance use trajectories, intentions, decision-making, and meaning-making processes (i.e., their thoughts, feelings, and identity-related struggles). The semi-structured format enabled participants to narrate their experiences and perspectives openly, facilitating the collection of rich data (39). The interview questions covered initiation and patterns of substance use, contexts and triggers, perceived effects and risks, concealment and disclosure, help-seeking, and reflections on professional identity, values, and practice.

Written informed consent was obtained from each participant, ensuring their full understanding of the voluntary nature of participation, their right to withdraw at any time, and the confidentiality of their data. As a token of appreciation for their participation, each participant was given HK$200 supermarket vouchers [US$25.6].

To preserve the fidelity of the data, all interviews were conducted in spoken Cantonese, audio-recorded, and transcribed. Each utterance was first converted from spoken Cantonese into written Cantonese (to standardize colloquial expressions) and then translated into English. Two bilingual researchers checked translations against the original Cantonese transcripts, and any discrepancies were discussed and resolved through consensus.

### 2.3 Analysis

The data were analyzed using reflexive thematic analysis (40) to identify, analyze, and report patterns/themes within the data that facilitate the understanding of participants’ experiences and stories. Analysis followed Braun and Clarke (40, p. 87) six-phase approach: (1) “familiarizing yourself with your data”; (2) “generating initial codes”; (3) “searching for themes”; (4) “reviewing themes”; (5) “defining and naming themes”; and (6) “producing the report”.

All transcripts were imported into NVivo to support data management and retrieval. Coding was conducted line-by-line and refined through iterative comparison across cases. Initial coding was primarily data-led and inductive, while subsequent interpretation was informed by concepts of cognitive dissonance and professional identity.

This study adopted an interpretivist paradigm and used reflexive thematic analysis to examine how the participants make sense of their experiences within the context of professional identity, stigma, and work-related demands. Analysis was iterative, with themes developed and refined through ongoing engagement with the coded data and reflexive interpretation. **Table 2** summarizes themes, subthemes, and illustrative excerpts.

**Table 2:** Summary of Themes and Subthemes.

Theme	Subtheme	Analytic focus	Illustrative excerpt
Overview of substance use trajectories	Initiation and patterns of substance use	Initiation, patterns of use, and the social, sexual, personal, and work-related circumstances surrounding substance use.	“Stress had reached a peak, and I went to a private den to use (substances) on my own.” (Participant 8)
Meanings of substance use	Alleviation, escape and stimulation	Substance use as escape/alleviation, stimulation and relief from mundane life.	“Having a couple of puffs is just taking a break - … one still needs a pause, right?” (Participant 13)
Rationalization and negotiation of professional identity	Compartmentalization	Separating substance use from professional roles through concealment, selective disclosure, self-imposed boundaries, and perceived maintenance of everyday functioning.	“It’s purely to prepare myself well … ‘Don’t get sick …’ I will talk to myself like that.” (Participant 1)
	Redefinition and Normalization of Substance Use	Reinterpreting substance use as common, manageable, personally meaningful, or less harmful than socially assumed in order to reduce perceived conflict with professional identity.	“When I first saw it, I really thought it was a drug. … when I saw it for the second time, I used it.” (Participant 3)
	Reframing Lived Experience as a Resource for Professional Practice	Framing personal substance-use experience as contributing to perceived empathy, non-judgmental understanding, credibility, and rapport with clients.	“I would be willing to share my personal experience and tell the audience how beneficial marijuana can be.” (Participant 1)
Views on abstinence and continued use	Abstinence, moderation, and life-course change	Abstinence and moderation as personalized choices shaped by motivations, relationships and life stage.	Temporary abstinence and moderation were commonly described as options rather than desistance.
Views on rehabilitation services	Experiences and perceptions of rehabilitation	Critical accounts of rehabilitation as rigid/old-fashioned and poorly aligned with nuanced motivations.	“Rehabilitation services were very old-fashioned, rigid and traditional.” (Participant 5)

Reflexivity was integral to the design and analysis. The research team acknowledged that prior academic and practice-based familiarity with substance use and helping professions could shape both interviewing and interpretation. Throughout the project, we kept reflexive notes and analytic memos (including within NVivo) to document emerging assumptions, alternative readings, and decisions about code/theme boundaries. Differences in interpretation were treated as analytic resources and were discussed in team meetings to strengthen the credibility and transparency of the analysis.

### 2.4 Ethical considerations

All procedures performed in the study adhered to the ethical standards of the Research and Ethics Committee of City University of Hong Kong. The confidentiality of the participants was maintained throughout the study by assigning pseudonyms. All data were stored on a secure, password-protected server to which only the research team had access.

## 3 Results

### 3.1 Overview of the helping professionals who engage in substance use

Among the reported substances, cannabis is the most commonly used. Only one participant indicated that he had never used cannabis. Regardless of their professional roles, their initial encounters with substances frequently revolved around social or sexual purposes and family conflict.

Participant 1 started using cannabis due to low tolerance to alcohol, which led to feeling unfulfilled in social gatherings. Due to the widespread perception of cannabis, 4 participants expressed an enduring interest in trying it. As such, they began using cannabis during social gatherings with peers who used substances or when visiting countries where its use was legal. They emphasized enjoying altered states of consciousness and bonding with friends over substances.

Men Who Have Sex with Men (MSM) participants used harder, more potent, and potentially more dangerous substances than other participants did. They often used substances in conjunction with their sexual contexts. They also relied on substance-using partners to judge whether a substance was safe to use. Despite knowing its potential for dependency, participant 2 initially underestimated this aspect of the substance and rationalized its use as a means of enjoyment and coping with emotional issues.

Participants exhibited variations in substance use frequency. However, 11 of them reduced their use after peaking. Only participants 5 and 6 continued to use substances after work daily, while the others reported using only on weekends or even once every few months. While 13 participants had no intention of quitting substances completely, only two were abstinent at the time of the interview: Participant 9 had maintained abstinence for three months, and Participant 3 for over three years.

### 3.2 Meanings of substance use

Substance use often served as a form of alleviation (escapism) to cope with personal problems. 7 participants believed that the substances helped them adopt an open-minded, easygoing attitude towards life events. The altered states of consciousness and minds after using substances unveiled a forbidden side of life and community, allowing participants to temporarily escape and alleviate life stressors, stigmas, and unfavorable emotions. Participant 7 highlighted that, as an MSM, substances provided an outlet for experiencing natural excitement and releasing emotions. The social judgments and stigma that sexual minorities face make them more vulnerable to the apparent effects of substance use, including stimulation and relief from mundane life.

Perhaps, societal judgment has been placed on my behavior or what I have done before. It is prevalent in our society, and many restrictions and norms are in place. However, I no longer feel constrained by these societal norms after taking substances. I am free to do whatever I want, even if I know that it is not socially acceptable or morally right. This newfound freedom can lead me to act in crazy ways that I wouldn’t normally consider. (Participant 2)

At that time, stress had reached a peak, and I went to a private den to use (substances) on my own. … Taking Ketamine is basically about trying to escape from the world in the long run. Cannabis, on the other hand, is meant for feeling happy and chilled out. (Participant 8)

Substances have also been depicted as a coping mechanism for emotional distress. Participant 5 described cannabis as a “good friend” during a period of “life’s low tide”, suggesting that the substance served as a temporary escape from the difficult reality while downplaying its harmful aspects.

Participants 2, 8, 11, and 15 reported having been diagnosed with depression. Some described worsening mood and perceptual experiences associated with substance use. During periods of deteriorating mental health, some also reported past suicide attempts. Despite these experiences, one participant continued to perceive controlled substance use as beneficial.

No participants directly mentioned experiencing burnout at work, but 5 participants described substance use as an alleviator. Participant 12 remarked that without at least a puff of cannabis after work every day, life pressures felt overwhelming. Participant 5 outlined the comforting effect of substance use on tough working days, while also acknowledging cannabis use as a “dependence” rather than a source of happiness. This ambivalence reflects the complex meaning of cannabis, suggesting a struggle between motivation and self-control. Participants 4 and 13 also cited using cannabis to unwind from work pressures but rejected the domination of substances in their lives.

For me, describing cannabis is like having a meal. Cannabis is never the main course, dessert, or appetizer—it’s just something extra, like a seasoning or condiment that adds flavor to the dish. (Participant 4)

Having a couple of puffs is just taking a break - even if one were to end one’s life, one still needs a pause, right? (Participant 13)

They viewed cannabis as a way to temporarily liberate themselves from societal demands, allowing them to relax and live in the moment. However, they also admitted the detrimental consequences of excessive use to cope with life problems instead of addressing the problems directly.

In summary, the meaning of substance use was complex for the participants. While they knew the potential harm of substance use and recognized the need to control this behavior and reduce its frequency, they (except for Participants 3 and 9) still chose to use substances because substances helped alleviate certain distress and stigma they experienced.

### 3.3 Strategies when facing cognitive dissonance: the use of rationalization for the conflicting roles

Helping professionals who engage in substance use face a conflict between their professional role and their own substance use. To navigate this tension, they employed various forms of rationalizations to justify their substance use while maintaining their professional identities. Several key rationalizations emerged from the participants’ narratives, allowing them to minimize the perceived harm of their substance use while maintaining a sense of control and balance in their lives. These rationalizations represent participants’ subjective understanding of substance use and should not be interpreted as the authors’ assessment of the relative pharmacological risks or safety of the substances discussed.

#### 3.3.1 Compartmentalization

A consistent thread across the narratives is the use of compartmentalization to keep social and professional lives separate. The social use of substances among 7 participants revolved around a small but trusted circle, avoiding judgmental or professional contacts. They concealed their use from colleagues and unsympathetic contacts to avoid judgment while admitting it to trusted friends.

Impacts on daily functioning are the key indicators of successful compartmentalization. Literature shows that most social workers tend to assess client substance use and dependence based on the client’s capability to fulfill responsibilities and perform daily functions rather than relying on practice guidelines or validated tools (41). Similarly, participants assessed their substance use and dependence through self-assessment of daily functions. As long as they perceived no influences on their job duties and fulfilled responsibilities, regardless of the actual impacts, they justified their substance use.

All the participants claimed that their substance use did not cause any physical illness or impair their capabilities. Perceiving minimal impacts on their lives and daily functioning, they rationalized their behaviors.

I might only do this thing once every three months, without affecting my life or other things. Actually, I don’t have any reason to completely stop doing it, as long as I can bear the consequences. For example, if I go playing for five or six hours and then finish what I want to do, I can continue to go to work. I only do it once every three months or half a year, so I don’t really need to stop. (Participant 2)

12 participants maintained functioning roles by self-imposing restrictions on substance use before work and moderating their intake. They maintained substance use at a personal level, creating a clear separation between their work and personal lives. However, deep down, 9 participants acknowledged a decline in their functioning. They reported experiencing mental exhaustion, emotional numbness, diminished workability and memory, depressive symptoms, and negative impacts on their life plans. Participant 2 specifically noted the lingering effects of substance use.

When you frequently use the substance, there will also be a lot of residual substances in your body and the environment. I felt it continued to affect me and had side effects accompanying me for a long time, maybe up to three to six months. Only when that time has passed can you really tell whether you are still being affected by the substance. (Participant 2)

This apparent discrepancy suggests that participants could maintain an overall perception of themselves as functioning adequately while simultaneously acknowledging specific adverse effects, reflecting the complexity of their self-assessment rather than a simple distinction between impaired and unimpaired functioning.

Despite recognizing the negative consequences, they continued to derive pleasure from certain effects, such as coping with life stress, releasing inhibitions, or achieving a certain mental state, reflecting a nuanced relationship with substances. Participant 1 even mentioned engaging in self-preparation to enhance enjoyment before substance use, but not for optimal job performance.

There is some anticipation for the upcoming use of substances. The night before, I will make sure to get enough sleep. I will sleep a bit more to fully charge myself. It’s purely to prepare myself well, like “Don’t eat too much junk food. You might feel uncomfortable later”. “Don’t get sick. If you do, you’ll feel uncomfortable and won’t want to go anywhere then”. I will talk to myself like that. (Participant 1)

In contrast, 5 participants did not perceive any negative impacts on their functioning. Rather, they perceived substances as enhancing their functioning. Participants 6 and 14 believed the pain-relieving effects of cannabis helped manage headaches and menstrual cramps, allowing them to maintain normal functioning. 3 participants reported that the substances enhanced their focus and energy for work.

It is noteworthy that participants who were medical professionals appeared to report fewer negative effects on their functioning. Participant 7 reported that he utilized his medical knowledge to examine the side effects of substances, including their duration, metabolic processes, and potential physical damage. Additionally, these participants leveraged their medical expertise to mitigate harm associated with substance use, such as Chinese Herbs. Notably, they revealed a method to speed up detoxification processes and avoid drug tests. Regardless of their effectiveness, the medical background indeed altered their perception of substance use and its dangers.

#### 3.3.2 Redefinition and normalization of substance use

Participants used various strategies to justify their substance use, revealing the complex relationship between societal perceptions, personal choice, and potential risks or benefits. Reframing illegal substance use as an act of free will or mature decision-making, they viewed it as a choice of lifestyle rather than a moral failing. However, some recognized the need for discretion and were aware of the stigma and legal barriers surrounding substances like cannabis. Ultimately, they navigated a delicate balance between using substances without judgment and exercising caution to avoid negative outcomes.

For 12 participants, the illegal status of certain substances (e.g., cannabis) was reframed as a political injustice rather than a reflection of inherent dangers. Participants 1, 6, 10, and 12 pointed out that the consequences of excessive consumption of tobacco, wine, and candy can be even worse than those of cannabis, highlighting the biased and unreasonable nature of current anti-drug policies in Hong Kong that still ban cannabis as an illegal, dangerous substance. 5 participants emphasized that as long as usage was controlled, substances, including cannabis and Meth, were merely medicines. By redefining illegal substances as “political injustices” rather than “inherent dangers”, participants justified their continued use as a considered personal choice rather than a moral failing, thus countering the societal stigmatization of substance users.

Participants normalized substance use by linking it to relaxation and creativity to destigmatize the use. Participants 4, 10, and 14 argued that responsible cannabis use was compatible with being a productive citizen, citing their own ability to function at work and the nonviolent nature of cannabis intoxication. Participant 6 linked cannabis use to self-care, practicing self-medication with substance use, and reconciling the stigma against cannabis. Participant 8 believed that people who smoked cannabis were very laid-back and had a sort of “utopian” mindset, and their friends who used marijuana or LSD were often designers seeking inspiration through marijuana use.

The redefinition was also facilitated by exposure to information. Adopting external justifications effectively handled the dissonance, such as seeking new information or selectively engaging with information that aligned with their beliefs (27). Participant 2 reported using the DSM-5 criteria to monitor symptoms. Once the symptoms were not observed, he personally deemed it safe to continue use. Participant 2 also argued that substances were unfairly stigmatized, as their use did not necessarily impede normal functioning, resembling legalized gambling. Thus, self-awareness mattered more than the prohibition of substances. Similarly, Participant 5 believed that using cannabis was normal and trivial based on episodes on Netflix:

When you see this [on Netflix], you realize that it is all based on research. Then, you understand that it is not a big deal. If you can afford to take on the risk, you may develop a new worldview and perspective on life. So why not give it a try? (Participant 5)

Participant 3 initially resisted using substances during sexual activity; however, pressure from romantic partners created a conflict between the desire to please them and personal beliefs about potential harm from substance use. A process of redefining the substance as less harmful, or even as potentially enhancing the romantic relationship, was then noted.

When I first saw it, I really thought it was a drug. Then, slowly, maybe because of the information online, I started to think it was very common. So, when I saw it for the second time, I used it. (Participant 3)

Thus, the dissonance arising from the conflict between the awareness of potential harm and the desire to seek pleasure or escape from emotional distress was resolved by downplaying the risks and emphasizing the benefits.

#### 3.3.3 Reframing lived experience as a resource for professional practice

13 participants described social stigmatization of substance use as a barrier for helping professionals to develop rapport and establish trust with service users. 5 participants further explained that judgmental attitudes obscure the true motivations behind substance use. Participant 3 proposed that ordinary secondary school teachers lacked the knowledge and rapport to recognize and handle substance use issues among students, leading to an inability to notice suspicious signs. Furthermore, because of the judgmental attitudes, students were reluctant to confide in them, creating mistrust between the two parties. Participant 3, using euphemisms to build rapport with students who potentially used substances, contended that the unique position as both a teacher and substance user allowed him to notice early signs of substance use and offer spaces for honest sharing, fostering trust.

Despite being unable to overtly disclose substance use, participants alluded to the perspective that it was not an unequivocal moral failing.

That being said, if I were to give a talk, I would be willing to share my personal experience and tell the audience how beneficial marijuana can be. (Participant 1)

It is more complex in Hong Kong … there are some grey areas where something may not truly be considered a substance and the like. In other words, different areas, different countries have different classifications of what is considered a drug. (Participant 3)

4 participants also proposed that modeling could help build trust with clients. As healthcare workers, participants 5 and 15 believed that their own substance use assisted in empathizing with youth struggling with substance use issues by demonstrating controlled use. Mature, mindful cannabis use was regarded as compatible with productivity, challenging stereotypes and emphasizing cannabis’s personal, therapeutic benefits rather than harm.

From a social worker’s perspective, it can be challenging to address the issue of substance use. However, I try to do what I can and speak to those around me, including my family. At times, I even tell my family that marijuana is not necessarily a bad thing and that taking action [to remove the stigmas towards marijuana] starts with oneself. (Participant 4)

Cannabis is legally used in many countries. As long as you can show you need emotional or psychological relief - or to deal with distress - I don’t understand why Hong Kong is so strict. This kind of relief is especially necessary in high-pressure places like Hong Kong. (Participant 15)

Hence, participants believed their substance use enhanced their empathy and understanding of users, facilitating better service to clients, especially youths.

When I provide counseling and speak with others, I often find that I already know many things. I can really use my heart to feel your heart—this is the goal of counseling. I’ve already walked this path once and understand all the experiences. (Participant 2)

In summary, by leveraging their unique insider perspectives as both professionals and users, participants could understand the clients’ motivations underlying substance use, developing an alternative form of trust. Participants perceived their experience as helping them open communication and establish rapport with clients.

### 3.4 Participants’ views on abstinence

13 participants believed that they did not need to stop using substances. Additionally, 12 participants had never received treatment. They regarded temporary abstinence and moderation as options rather than desistance. Participant 7 received a probation order that required quitting substances and occupied their time with exercise and hobbies. However, upon release, they intended to moderate their use rather than fully stop and to participate in healthier programs. For 3 female participants, pregnancy emerged as a potential circumstance for temporary abstinence. They were uncertain about whether they would maintain abstinence after giving birth. For instance, participant 9 admitted to occasional substance use during pregnancy and after giving birth.

Only two participants remained abstinent. Participant 3 viewed recreational substances as unnecessary crutches after finding meaningfulness through an emotional connection in an intimate relationship. Participant 3 originally used poppers for social reasons, implying the importance of emotional connections. Over time, it became clear that poppers solely facilitated superficial relationships that could not fulfill their needs. Eventually, participant 3 stopped using substances after starting a committed relationship, signifying a loss of appeal and the meaningfulness of substance use. For participant 9, abstinence was inspired by her mother’s liver cancer, promising to become a doctor who could heal others. Additionally, upon self-reflection, there was hope that their child would not repeat the “mistakes”.

### 3.5 Participants’ views on rehabilitation services

Some participants found rehabilitative services ineffective and impersonal for achieving abstinence. Having received traditional treatment, participant 7 believed that treatment should be tailored to individual needs. 3 participants even used words like “mental breakdown” and “suicidal” to describe the rehabilitation service.

The rehabilitation services were completely useless. Being there pushed me into a mental breakdown and made me question why I was even present. They simply locked me up, making me feel even more desperate and suicidal. (Participant 9)

I really do not understand why they had to treat me like a military camp. I also do not know why that would help me quit gambling or substances. It was a painful experience … No one cared about me; no one cared about how I felt. (Participant 13)

Similarly, participant 5 commented that rehabilitation services were very old-fashioned, rigid, and traditional. Their responses underscored abstinence and moderation as highly personal choices driven by individual motivations and usage patterns. Blanket approaches failed to address the nuanced reasons behind their use and “individuals’ preferences” (42, p. 12). Ultimately, understanding these motivations and lifestyles emerged as the key to supporting meaningful reduction or cessation of problematic use.

## 4 Discussion

This study explored substance use among helping professionals as an identity-management process shaped by cognitive dissonance and organizational stigma. Drawing on the interviews with Hong Kong helping professionals across various settings, the data suggest how they sustained professional legitimacy through three recurrent strategies: (1) compartmentalizing substance use from professional roles, (2) redefining or normalizing the meanings and perceived risks of substance use, and (3) framing lived experience as potentially valuable for empathy and trust-building with clients. These findings offer preliminary, context-specific insight into how dissonance management may be shaped by professional norms, stigma, and organizational contexts, and they highlight the need for confidential, non-punitive, and context-sensitive support pathways for practitioners.

While past research has attributed helping professionals’ substance use to job strain (e.g., 5, 8, 30), participants in the present study did not explicitly describe burnout as a reason for their substance use. Nevertheless, some described substance use as a means of unwinding from work-related pressure. Work stress, therefore, appeared as one contextual factor, although the data do not establish it as the primary cause of substance use. This implies that the meanings and circumstances surrounding substance use may extend beyond work-related factors and may also involve personal, social, and other life experiences.

Additionally, previous research suggests that substance use may also precede entry into helping professions (16), reinforcing the need not to attribute substance use solely to occupational stress, such as adverse personal life events (43). On the other hand, results uncovered how the professionals perceive their substance-using behaviors. In the literature, social workers who use substances perceived such behaviors as enhancing their work with clients facing similar issues (22). From their lens, substance use is viewed as a way for professionals to build trust with clients, essential for positive outcomes (44, 45). Those with lived experience can enhance this trust, helping clients feel more comfortable and be honest (44). Participants in this study shared similar perspectives, drawing on their experiences to better address their clients’ needs and enhance their roles in the treatment and rehabilitation. They also linked their lived experiences to a more empathetic, non-judgmental perspective on clients, which they believed enhanced empathy and rapport in their professional interactions. This supports previous research that professionals’ lived experiences with challenges (e.g., mental health issues, addictive behaviors) help them connect empathically with the clients (e.g., 7, 16, 22).

Meanwhile, the lived experiences of the participants displayed profound dissonance between their personal and professional lives, struggling to balance their professional responsibilities with substance use. Despite acknowledging associated risks, they rationalized their behaviors using three strategies: (1) compartmentalizing their social and professional lives; (2) redefining the risks and illegal status; and (3) arguing that substance use facilitated alternative trust-building with clients. They regarded their continued use as a conscious personal choice rather than a lack of control. While some studies suggest that substance use does not necessarily impair professionals’ work performance (46), some participants described substance use in ways that suggested escape-oriented coping with distress. Although their substance use primarily serves recreational, social, and stress-relief purposes, the line between alleviation and escapism is not always clear, implying a more dysfunctional, escape-oriented use. While their insider knowledge and openness about substances are generally perceived as not interfering with their daily functioning and professionalism, which may provide justification for their behavior, they may not realize that their self-assessment of substance use could be inaccurate (41), reflecting their need for service support.

The findings highlight how prevailing professional impairment discourse, often embedded in organizational policies, professional standards, and supervisory cultures, can unintentionally intensify secrecy and delay help-seeking among helping professionals who use substances. When substance use is framed primarily as fitness-to-practice failure, practitioners may anticipate punitive consequences (e.g., loss of employment, registration jeopardy, disciplinary investigation), thereby favoring concealment, compartmentalization, and informal self-management over seeking timely support (e.g., 47, 48). This dynamic is particularly salient in settings where workplace substance testing is implemented as a compliance tool, as it may deter disclosure and early intervention, pushing problems underground rather than reducing risk (e.g., 49). Similarly, mandatory reporting, whether formalized in law, organizational policy, or professional culture, can generate a perceived no-safe-pathway for confidential assistance, especially where reporting thresholds are unclear or interpreted conservatively (47, 50). Such institutional responses risk producing perverse effects by eroding workforce well-being through the amplification of stigma and fear, while also discouraging proactive support-seeking, reflective supervision, and organizational learning, ultimately undermining service quality (e.g., 47). A “zero tolerance” risk-management approach can create a governance paradox: the more focus on detection and sanction, the less likely practitioners are to disclose early or seek support (51, p. 1880), increasing downstream risks to workforce wellbeing and service quality. Therefore, a more balanced governance approach, which differentiates substance use from demonstrable practice-related impairment, establishes clear and proportionate escalation pathways, and offers confidential, non-punitive supports while ensuring client safety, is needed.

## 5 Limitations of study and future research

This study had a limited sample size, which limits transferability beyond this sample and context. This exploratory qualitative study, centered on an under-researched population, presents findings that reflect the subjective experiences, interpretations, and meaning-making among the substance-using helping professionals. The study sought to foreground participants’ subjective understanding and interpretation of their experiences within their social and professional contexts.

Yet, several limitations should be considered when interpreting the findings. First, participation was voluntary in a sensitive area, which may mean those who disclosed their substance use differ from non-participants. Second, the findings are based on retrospective, self-reported accounts that solely reflect subjective interpretations. Third, the sample included various helping professions with different substance-use patterns, and the study did not aim to make occupation- or substance-specific comparisons. Fourthly, participants’ accounts of professional functioning were not independently triangulated through colleagues, supervisors, work records, or other objective indicators; accordingly, the findings concern perceived rather than objectively verified functioning. These limitations should be kept in mind when interpreting participants’ accounts.

Nonetheless, this study highlights the significance of addressing this issue and calls for further research to develop tailored interventions and effective policies for substance-using helping professionals. Future research could explore how personal substance use affects counseling approaches, the impact of substance-related regulations on them, and the effectiveness of various harm reduction strategies for this population. It would also be beneficial to consider these topics in larger, multi-site, or mixed-methods studies that incorporate diverse data sources.

## 6 Conclusions

This study provides insight into the lives of helping professionals who are also substance users. It reveals the complex interplay among their roles, justifications, resolutions, and psychological states, underscoring the importance of understanding these dynamics for developing effective strategies. A comprehensive and empathetic approach is essential in addressing this issue.

Regarding service implications, the findings underscore the importance of developing a more nuanced and individualized intervention that take account of helping professionals’ motivations, perceived functioning, and concerns about disclosure (5). Traditional rehabilitation programs, featuring medication, education, and therapies to manage withdrawal symptoms, prevent relapses, and restore functions (e.g., 52, 53), centered primarily on abstinence may not fully address the varied meanings and circumstances surrounding their substance use. Harm-reduction-informed approaches, which seek to reduce substance-related harms rather than making abstinence the sole objective (54), may therefore warrant further evaluation for this population (52). Such approaches could include access to risk-reduction information and practices, recognition of warning signs of problematic use or dependence, and strategies for managing stress (e.g., meditation) and other circumstances associated with substance use (55). Support addressing underlying personal or contextual difficulties may also be relevant, consistent with calls for interventions to engage with the “root causes” of substance use (56, p. 241). In addition, safe, confidential and stigma-reducing forms of support, including in-depth counselling, mutual support groups, and supportive, non-retributive measures, could be explored as ways of facilitating earlier help-seeking while respecting professionals’ concerns about disclosure and professional consequences (13, 22, 45, 57).

Regarding policy and institutional implications, the findings suggest that alternatives to exclusively punitive or “zero-tolerance” responses are necessary. A harm reduction-informed workforce approach could place greater emphasis on early support, confidentiality, proportionate responses, and the distinction between substance use and demonstrable practice-related impairment, while maintaining appropriate safeguards for service users. This aligns with international workplace guidance emphasizing prevention, treatment/rehabilitation, and confidentiality rather than automatic punishment (58). Operationally, policies could include clear thresholds distinguishing substance use from demonstrable practice-related impairment, confidential disclosure routes (e.g., confidential EAP/occupational health access before formal escalation), and supportive return-to-work pathways with structured monitoring — consistent with evidence-based physician health program models (58).

## Data Availability

The datasets presented in this article are not readily available because of the sensitive nature of the topic and the risk of participant identification. Additional data may be made available to qualified researchers upon reasonable request, subject to ethics approval and a data-sharing agreement. Requests to access the datasets should be directed to Prof. T. Wing Lo, lotitwing@gmail.com.

## References

[1] Narcotics Division, Security Bureau . Central Registry of Drug Abuse: Seventy-Fourth Report 2015 - 2024 (2025). Available online at: https://www.nd.gov.hk/pdf/report/crda_74th/CRDA_74th_Report_Full_Version.pdf (Accessed March 1, 2026).

[2] Cheng CP . Psychiatrists: Cases of Medical Professionals Abusing Drugs Increased; Relieving Stress Before Work. HK01 (2019). Available online at: https://www.hk01.com/article/304154?utm_source=01articlecopy&utm_medium=referral (Accessed March 1, 2026).

[3] Chiu YLM . A Survey on the Stress and Mental Health of Frontline Social Workers: A Summary (2006). Available online at: https://www.legco.gov.hk/yr05-06/chinese/panels/ws/papers/ws0612cb2-2397-3c-scan.pdf (Accessed March 2, 2026).

[4] Shy BD Portelli I Nelson LS . Emergency medicine residents’ use of psychostimulants and sedatives to aid in shift work. Am J Emerg Med. (2011) 29:1034–1036.e1. doi: 10.1016/j.ajem.2010.06.004 20708878

[5] Waite-Labott K . Substance Use Disorder in Healthcare Professionals: When Caregivers Need Care and Treatment. Switzerland AG: Springer Nature (2022).

[6] Kiepek N Harris J Beagan B Buchanan M . Substance use by social workers and implications for professional regulation. Drug Alcohol Today. (2019) 19:147–59. doi: 10.1108/DAT-08-2018-004035579975

[7] Newcomb M Burton J Edwards N . Service user or service provider? How social work and human services students integrate dual identities. Soc Work Educ. (2017) 36:678–94. doi: 10.1080/02615479.2017.132757437339054

[8] Stelson EA Sabbath-Clayton LL Sorensen G Kubzansky LD Berkman LF Sabbath EL . Residential addiction treatment providers: identifying the role of social context in worker health and turnover. Soc Sci Med. (2022) 314:115462. doi: 10.1016/j.socscimed.2022.115462 36327634

[9] Kunyk D . Substance use disorders among registered nurses: prevalence, risks and perceptions in a disciplinary jurisdiction. J Nurs Manag. (2015) 23:54–64. doi: 10.1111/jonm.12081 23952722

[10] Pooler D Sheheen F Davidson J . Professional impairment: a history and one state’s response. J Addict Dis. (2009) 28:113–23. doi: 10.1080/10550880902772415 19340673

[11] Brooks SK Gerada C Chalder T . Review of literature on the mental health of doctors: are specialist services needed? J Ment Health. (2011) 20:146–56. doi: 10.3109/09638237.2010.541300 21275504

[12] Cares A Pace E Denious J Crane LA . Substance use and mental illness among nurses: workplace warning signs and barriers to seeking assistance. Subst Abus. (2015) 36:59–66. doi: 10.1080/08897077.2014.933725 25010597

[13] Warren L Feit MD Wells J . Substance abuse among professionals: limited research on substance-abusing social workers. J Hum Behav Soc Environ. (2011) 21:803–12. doi: 10.1080/10911359.2011.61675337339054

[14] Bryson EO . The opioid epidemic and the current prevalence of substance use disorder in anesthesiologists. Curr Opin Anesthesio. (2018) 31:388–92. doi: 10.1097/ACO.0000000000000589 29474213

[15] Oreskovich MR Shanafelt T Dyrbye LN Tan L Sotile W Satele D . The prevalence of substance use disorders in American physicians. Am J Addict. (2015) 24:30–8. doi: 10.1111/ajad.12173 25823633

[16] Straussner SLA Senreich E Steen JT . Wounded healers: a multistate study of licensed social workers’ behavioral health problems. Soc Work. (2018) 63:125–33. doi: 10.1093/sw/swy012 29425335 PMC6042294

[17] Rojas JI Brand M Fareed S Koos E . Psychiatric comorbidity in health care professionals with substance use disorders. Addict Disord Their Treat. (2013) 12:51–7. doi: 10.1097/ADT.0b013e318250a3d242585464

[18] Maslach C . The Cost of Caring. Englewood Cliffs, NJ: Prentice-Hall Inc (1982).

[19] Merlo LJ Trejo‐Lopez J Conwell T Rivenbark J . Patterns of substance use initiation among healthcare professionals in recovery. Am J Addict. (2013) 22:605–12. doi: 10.1111/j.1521-0391.2013.12017.x 24131169

[20] Baldisseri MR . Impaired healthcare professional. Crit Care Med. (2007) 35:S106–16. doi: 10.1097/01.CCM.0000252918.87746.96 17242598

[21] Arble E Manning D Arnetz BB Arnetz JE . Increased substance use among nurses during the COVID-19 pandemic. Int J Environ Res Public Health. (2023) 20:2674. doi: 10.3390/ijerph20032674 36768040 PMC9915151

[22] Steen JT Cooper C . I had to fix me”: social workers and substance misuse. J Soc Work Pract Addict. (2022) 22:344–9. doi: 10.1080/1533256X.2022.208427637339054

[23] Brotherton S Kao A Crigger BJ . Professing the values of medicine: the modernized AMA code of medical ethics. JAMA. (2016) 316:1041–2. doi: 10.1001/jama.2016.9752 27415447

[24] Denzin NK . A phenomenology of the emotionally divided self. In: Yardley K Honess T , editors. Self and Identity: Psychosocial Perspectives. John Wiley & Sons Ltd, Chichester (1987). p. 287–96.

[25] Festinger L . A Theory of Cognitive Dissonance. Stanford, CA: Stanford University Press (1957).

[26] Eiroa-Orosa FJ Gebac S Braquehais MD Llavayol E Garcia-Gutierrez A Feixas G . Substance abuse among health professionals: a personal construct analysis. J Constr Psychol. (2019) 34:463–78. doi: 10.1080/10720537.2019.170667637339054

[27] McGrath A . Dealing with dissonance: a review of cognitive dissonance reduction. Soc Pers Psychol Compass. (2017) 11:e12362. doi: 10.1111/spc3.1236240046247

[28] Beisecker AE . Interpersonal communication strategies to prevent drug abuse by health professionals and the elderly: contributions of the health belief model. Health Commun. (1991) 3:241–50. doi: 10.1207/s15327027hc0304_642832780

[29] American Addiction Centers . Drug & Alcohol Rehab for Healthcare Professionals Near You (2023). Available online at: https://americanaddictioncenters.org/healthcare-professionals (Accessed March 1, 2026).

[30] Oreskovich MR Kaups KL Balch CM Hanks JB Satele D Sloan J . Prevalence of alcohol use disorders among American surgeons. Arch Surg. (2012) 147:168–74. doi: 10.1001/archsurg.2011.1481 22351913

[31] Zerubavel N Wright MO . The dilemma of the wounded healer. Psychother. (2012) 49:482–91. doi: 10.1037/a0027824 22962968

[32] Worley J . Nurses with substance use disorders: where we are and what needs to be done. J Psychosoc Nurs Ment Health Serv. (2017) 55:11–4. doi: 10.3928/02793695-20171113-02 29194558

[33] Baumgart-McFarland M Chiarello E Slay T . Reluctant saviors: professional ambivalence, cultural imaginaries, and deservingness construction in naloxone provision. Soc Sci Med. (2022) 309:115230. doi: 10.1016/j.socscimed.2022.115230 35995012

[34] Daibes MA Al-Btoush MS Marji T Rasmussen JA . Factors influencing nurses’ attitudes towards patients in Jordanian addiction rehabilitation centres: a qualitative study. Int J Ment Health Addict. (2017) 15:588–603. doi: 10.1007/s11469-016-9682-230311153

[35] Earnshaw V Smith L Copenhaver M . Drug addiction stigma in the context of methadone maintenance therapy: an investigation into understudied sources of stigma. Int J Ment Health Addict. (2013) 11:110–22. doi: 10.1007/s11469-012-9402-5 23956702 PMC3743126

[36] van Boekel LC Brouwers EPM van Weeghel J Garretsen HFL . Stigma among health professionals towards patients with substance use disorders and its consequences for healthcare delivery: systematic review. Drug Alcohol Depen. (2013) 131:23–35. doi: 10.1016/j.drugalcdep.2013.02.018 23490450

[37] Narcotics Division, Security Bureau . Multi-Modality Approach to Drug Treatment and Rehabilitation (2024). Available online at: https://www.nd.gov.hk/en/treatmentandrehabilitation.html (Accessed March 1, 2026).

[38] Cheng KKY Ling HWH Chui WH . The future of Hong Kong’s anti-drugs campaign: Prospective practitioners’ views on engaging in drug abuse work, attitudes toward young drug users and satisfaction with drugs education. In: Van Hout MC , editor. Drug Use and Abuse: Signs/Symptoms, Physical and Psychological Effects and Intervention Approaches. Nova Science Publishers, Inc, New York (2013). p. 49–64.

[39] Anderson C Kirkpatrick S . Narrative interviewing. Int J Clin Pharm. (2016) 38:631–4. doi: 10.1007/s11096-015-0222-0 26613739

[40] Braun V Clarke V . Using thematic analysis in psychology. Qual Res Psychol. (2006) 3:77–101. doi: 10.1191/1478088706qp063oa

[41] Galvani S Hutchinson A Dance C . Identifying and assessing substance use: findings from a national survey of social work and social care professionals. Br J Soc Work. (2014) 44:1895–913. doi: 10.1093/bjsw/bct033

[42] Mahboub N Honein-AbouHaidar G Rizk R de Vries N . People who use drugs in rehabilitation, from chaos to discipline: advantages and pitfalls: a qualitative study. PloS One. (2021) 16:e0245346. doi: 10.1371/journal.pone.0245346 33544758 PMC7864414

[43] Lee GP Storr CL Ialongo NS Martins SS . Association between adverse life events and addictive behaviors among male and female adolescents. Am J Addict. (2012) 21:516–23. doi: 10.1111/j.1521-0391.2012.00285.x 23082829 PMC3481175

[44] Brekke E Lien L Biong S . Experiences of professional helping relations by persons with co-occurring mental health and substance use disorders. Int J Ment Health Addict. (2018) 16:53–65. doi: 10.1007/s11469-017-9780-9 29491766 PMC5814541

[45] O’Brien TC Feinberg J Gross R Albarracín D . Supportive environments during the substance use disorder epidemic in the rural United States: provider support for interventions and expectations of interactions with providers. Soc Sci Med. (2022) 294:114691. doi: 10.1016/j.socscimed.2021.114691 35033798 PMC12475983

[46] Kiepek N Beagan B Harris J . A pilot study to explore the effects of substances on cognition, mood, performance, and experience of daily activities. Perform Enhanc Health. (2018) 6:3–11. doi: 10.1016/j.peh.2018.02.00342574925

[47] Elwyn R . Fear of the impaired practitioner, mandatory reporting, and clinician suicide: a lived experience of fitness to practice investigation as a student. Disabil Stud Q. (2024) 43. doi: 10.18061/dsq.v43i3.8300

[48] Maben J Hoinville L Querstret D Taylor C Zasada M Abrams R . Living life in limbo: experiences of healthcare professionals during the HCPC fitness to practice investigation process in the UK. BMC Health Serv Res. (2021) 21:839. doi: 10.1186/s12913-021-06785-7 34412640 PMC8375211

[49] Banja J . Alcohol and drug testing of health professionals following preventable adverse events: a bad idea. Am J Bioeth. (2014) 14:25–36. doi: 10.1080/15265161.2014.964873 25369412

[50] Bismark MM Morris JM Clarke C . Mandatory reporting of impaired medical practitioners: protecting patients, supporting practitioners. Intern Med J. (2014) 44:1165–9. doi: 10.1111/imj.12613 25442757 PMC4313682

[51] Monahan G . Drug use/misuse among health professionals. Subst Use Misuse. (2003) 38:1877–81. doi: 10.1081/JA-120024245 14582582

[52] Gilbert AR Hellman JL Wilkes MS Rees VW Summers PJ . Self-care habits among people who inject drugs with skin and soft tissue infections: a qualitative analysis. Harm Reduct J. (2019) 16:69. doi: 10.1186/s12954-019-0345-z 31831010 PMC6909440

[53] McKay JR . Traditional approaches to addiction treatment and continuing care. In: Treating Substance Use Disorders With Adaptive Continuing Care. American Psychological Association, Washington, DC (2009). p. 35–61.

[54] Marlatt GA Witkiewitz K . Harm reduction approaches to alcohol use: health promotion, prevention, and treatment. Addict Behav. (2002) 27:867–86. doi: 10.1016/S0306-4603(02)00294-0 12369473

[55] Dartey AF Tackie V Inemesit BO Johnson BB Lotse CW Geraldo S . The challenges faced by medical personnel misusing drugs: a phenomenological study. SAGE Open. (2025) 15. doi: 10.1177/21582440251326348

[56] Kiepek N Beagan B . Substance use and mental health among Canadian Social Workers. Health Soc Work. (2024) 49:235–43. doi: 10.1093/hsw/hlae026 39265990

[57] Smith AM Campbell DG . Strategies to address substance-abusing and substance-impaired healthcare professionals. Int J Appl Manag Technol. (2020) 19:97–106. doi: 10.5590/IJAMT.2020.19.1.0839681816

[58] International Labour Organization . Management of Alcohol- and Drug-Related Issues in the Workplace: An ILO Code of Practice. Geneva: International Labor Office (1996).

